# The Control Strategies for Information Asymmetry Problems Among Investing Institutions, Investors, and Entrepreneurs in Venture Capital

**DOI:** 10.3389/fpsyg.2020.01579

**Published:** 2020-07-14

**Authors:** Peng Du, Hong Shu, Zhuqing Xia

**Affiliations:** ^1^School of Economics and Management, Shaanxi University of Science and Technology, Xi’an, China; ^2^School of Sciences, Chang’an University, Xi’an, China; ^3^Maolong International Financial Leasing Co., Ltd., Xi’an, China

**Keywords:** venture capital, information asymmetry, entrepreneurial psychology, investment efficiency, investing institution

## Abstract

To analyze the problem of information asymmetry between investment institutions, investors, and entrepreneurs, thereby protecting the interests of all parties and ensuring the income under high risks, some companies listed on Shanghai Stock Exchange and Shenzhen Stock Exchange are taken as research samples. First, the relations between investment institutions, investors, and entrepreneurs, as well as the causes and solutions of information asymmetry in venture capital, are analyzed. Second, based on the residual model, a model that analyzes the information asymmetry and investment efficiency of the enterprise is built. Finally, through two cases, the relation between the information asymmetry and the investment rate is revealed. The research results show that from 2014 to 2018, the investment level of companies listed on the Shanghai Stock Exchange and Shenzhen Stock Exchange was below 7.69%; the investment levels of different companies varied greatly, with significant differences in the development situations. Besides, information asymmetry is significantly related to investment efficiency. The corresponding regression coefficient is 0.0119. The larger the enterprise is, the higher the investment efficiency will be. The case analysis shows that it is important to understand and seize the market, and the information asymmetry between the actual environment and the investment environment is more serious. To deal with these issues, material incentives, reputation incentives, control incentives, and stock option incentives are substantive measures that can be taken, which are of great significance for improving the decision-making level of investors and investment institutions and reducing the investment risk of investors and entrepreneurs.

## Introduction

With the rapid development of traditional industries and the information industry, the concept of “big data” has begun to play a role in people’s daily lives. In the meantime, information economics has also been developed rapidly due to the development of big data, making information asymmetry widely concerned (Chen et al., 2017). In modern economic theory, the development of information economics is changing the traditional microeconomics and rebuilding the foundation of the entire economics. Generally, in all the interest-related trading activities, problems with information exist. In other words, the decision-making of transactions is generally based on incomplete and asymmetric information. This asymmetric information and its impact are more prominent in the capital market. In the trading behavior of the capital market, there is often a huge conflict of interest, and asymmetric information provides opportunities for fraud and unethical behavior in pursuit of interest. The problem of information asymmetry has become the focus of many researches. Under ideal circumstances, the ability to invest institutions, funders, and entrepreneurs in venture capital to obtain information is the same. As a result, information asymmetry does not exist (Kardan et al., 2016). However, there are differences among the three parties in the ability to obtain information, and they have different directions and major concerns about information collection. Therefore, the information asymmetry truly exists, which will become a factor that will bother the three parties (Han and Zhang, 2016). In addition, the weakening of information asymmetry and even its solution is the focus of attention of all sectors of society.

In China, the information asymmetry is a very serious problem. For investors, the system of the Chinese stock market is misplaced. Investors are in a disadvantaged position for there are relatively few channels to protect their interests. Thus, the risks are amplified further (Samet and Jarboui, 2017). There is an inevitable phenomenon of information asymmetry in venture capital, and it will have a very important impact on the efficiency of venture capital. In this way, how to effectively avoid the investment risk caused by information asymmetry has become a major factor that must be considered in the process of venture capital activities of major venture capital institutions (O’Toole et al., 2016). What entrepreneurs should do is to screen and use the information to reduce the problem of information asymmetry for investors. Therefore, this study discusses the control strategies for the problem of information asymmetry among investing institutions, investors, and entrepreneurs.

Although scholars from various countries have explored the problem of information asymmetry, there are very few works related to information asymmetry under China’s national conditions. To deal with such a situation, a series of measures could be taken. First, the information asymmetry and its cause in venture capital were described. Then, a detailed solution to the information asymmetry problem was proposed. Based on the previous researches, a research model was determined for the situation of information asymmetry affecting the investment efficiency of enterprises. Also, the losses caused by information asymmetry and the slow development of solutions were analyzed.

## Literature Review

There are a variety of studies on the information asymmetry among investing institutions, funders, and entrepreneurs in venture capital. Yong (2012) conducted a simulation study on the issue of information asymmetry and found that investors would choose the method of average valuation for investment activities in the case of asymmetric information, which would affect the development of some enterprises with high demand for cash flow; however, for enterprises with low demand for cash flow, over-investment would occur (Yong, 2012; Abidoye et al., 2019). d’Andria (2019) focused on the information asymmetry in the credit and labor markets at the same time. Considering the learning over time and its impact on tax and subsidy policies, simulation analysis was utilized to test the impact of tax policies on the entrepreneurs and subsidies on labor incomes (d’Andria, 2019). Based on the causality test of repurchase information and enterprise earnings, Lee and Mauck (2018) connected the informed repurchase to the asymmetry of corporate information and proposed a new informed repurchase measure. The results showed that when the repurchase of stocks in the open market was announced, a large abnormal return would be gained (Lee and Mauck, 2018). Asongu and Odhiambo (2018) investigated whether information sharing channels aimed at reducing information asymmetry had led to an increase in financial access. This study used generalized moment technology and data from 53 African countries from 2004 to 2011. To test this connection, the study found that the two types of information sharing channels, private credit agencies and public credit agencies, had a positive and significant impact on financial visits (Asongu and Odhiambo, 2018). Bushee et al. (2018) researched the relationship between language complexity and information asymmetry. In the quarterly earnings conference calls, the estimated information and fuzziness were two potential components. The results showed that the estimation of the information portion and the information asymmetry was in negative correlation, while the estimation of fuzziness is positively correlated with information asymmetry (Bushee et al., 2018).

As a provider of information, financiers have complete information, but for investors, the understanding of the company is often restricted by many aspects, and the use of information will also affect the behavior of investors. For example, when sufficient information is available, investors can continuously screen and refine useful information to make effective investment decisions. Research has pointed out that as investors, the understanding and judgment of the enterprise is mainly through the financial information disclosed by the listed enterprises. Due to the imperfect information environment of China’s disclosure mechanism and the lack of corresponding punishment, listed enterprises often have stronger motivation to obtain high valuation of financing offers for selective disclosure of information. In this case, the authenticity of the information obtained by investors will be greatly reduced, which will cause blind investment and lead to inefficient investment of enterprises (Chen et al., 2011). Due to the existence of information asymmetry, investors continue to realize that there is often untrue information in the markets they may contact, while they cannot discern the true information from the disclosed information. In this case, external investors often average the valuation of all enterprises, and this average evaluation will inevitably bring about the problem of adverse selection. In other words, those enterprises whose investment projects are below the average will benefit from the high valuation of investors, which will cause more such investment projects to enter the enterprises (Zhao and Song, 2018).

By summarizing previous results, it is found that the researches mainly discuss investment decisions and financing methods in a sound capital market, which is simply not applicable in China. Therefore, the situation where information asymmetry affects the investment efficiency of enterprises is explored. By analyzing the situation where information asymmetry leads to losses, this study proposes corresponding solutions to the venture capital problem in China’s financial market.

## Materials and Methods

### Information Asymmetry in Venture Capital

In general, information asymmetry is used to describe the differences in information obtained by different individuals during the transaction. Information asymmetry occurs frequently in daily life. Information asymmetry occurs in most social activities. This results in that those with more information are in an advantageous position in trading activities, while those with less information are in a disadvantaged position (Gan, 2019).

Information asymmetry also frequently occurs in venture capital, and it is the root of a series of practical problems in venture capital (Melzer, 2017). Due to the existence of information asymmetry, investors’ investment thinking and investment strategies are seriously affected, resulting in a great reduction in the benefits gained; thus, the problem of information asymmetry is also the most critical factor for investment errors.

Due to the high returns and high-risk nature of venture capital, the consequences of the information asymmetry problem in it will be far greater than those in other industries. The major information asymmetry problems in venture capital will exist in the following areas:

(1) Information asymmetry between the investors and the investing institutions: The investors need to invest their funds in the enterprises, and the professionals in the investing institutions need to manage the risks for investors; hence, the agency relationship between the two parties appears. The investing institutions possess professional financial knowledge, which materializes the risks generated by the investment process and thus helps investing institutions choose the distribution method of benefits and ensure that the benefits of the terms in the contract are maximized. However, investing institutions often fail to fully understand the investment process of investors. In the later period, human factors cannot be accurately considered, and the investing institutions cannot fully understand the specific investment strength of the investors, the strength of the investors, and the credit status. Therefore, this has resulted in information asymmetry between the investors and the investing institutions (Anifowose et al., 2018).
(2) Information asymmetry between investors and entrepreneurs: Investment agencies usually communicate and coordinate with investors, and the information asymmetry between investors and entrepreneurs often has the lowest adverse effects on investment.
(3) Information asymmetry between entrepreneurs and investing institutions: For entrepreneurs and investing institutions, entrepreneurs know better about the current status of the industry, rigid demand, profitability, and their own business methods and operating conditions. Therefore, the entrepreneurs are more aware of the income status of the investment projects, while the investing institutions cannot fully understand the use of funds and the human factors of the enterprises. This leads to information asymmetry between investing institutions and entrepreneurs. Most of the enterprises invested are emerging enterprises (Du et al., 2018). The records of such enterprises’ systems, credits, and finances are not comprehensive, leading to more serious information asymmetry between entrepreneurs and investing institutions. For investing institutions, each service staff member has advantages and disadvantages. Thus, it is impossible to determine whether the service staff member is best for the investment project.

### Causes of Information Asymmetry

There are many reasons for information asymmetry. This study will analyze the four aspects of investment characteristics in detail, including entrepreneurial psychology, limited rationality, and investment costs.

(1) Investment characteristics: The ultimate goal pursued in venture capital is high returns, and a variety of uncertainties in venture capital are the source of high returns. Therefore, new industries and high-tech industries tend to become the main investment objects of venture capital. Meanwhile, the lack of technical knowledge and industry knowledge has become the major cause of information asymmetry (Hao and Lu, 2018). The incomplete disclosure of entrepreneurs’ research results in an incomplete understanding of investors on the business of the entrepreneurs. Also, the insignificance of the impact of the risks to investors on the enterprises and the supremacy of corporate interests have led to increasingly serious information asymmetry.
(2) Entrepreneurial psychology: For entrepreneurs, whether or not to obtain investment is the most important way of thinking. As a result, in order to obtain the largest amount of investment, entrepreneurs may get a larger amount of investment through a series of packaging activities. Instead, investing institutions generally do not track the operating status of the enterprises in detail but rather determine whether to invest and the amount of investment through evaluation. Taking the entrepreneurial psychology as the starting point, if the current situation of the enterprise is not good, it will be better to get investor funds; if the entrepreneurs fail to get the funds, their existing funds will have no damages; if the entrepreneurs get the funds, they will have more liquid assets, which is conducive to the development of the enterprise. Therefore, the self-interest of entrepreneurs and risk-avoidance psychology become the major reasons for information asymmetry (Cheng et al., 2013).
(3) Limited rationality: Due to the deficiency of human physiology, psychology, and information cognition, everyone has limited rationality. Because of the continuous development of the industry, the knowledge and awareness of entrepreneurs in the industry are also in a state of limited rationality. For investing institutions, the professional knowledge of professionals and their understanding of the industry are also in a state of limited rationality. The psychological factors of managers themselves are also important reasons for the information asymmetry. Stronger self-confidence will lead the manager to be too optimistic about the risks and overly trust his own experience, ignoring the impact of the market and human factors on corporate profitability (Chen et al., 2018).
(4) Investment cost: In the current data world, data have become a new type of resource, so the cost of information collection has risen rapidly. The owner of the information sells it for the benefit of the information, and the rarity of the information also directly determines the price of the information. The information that investing institutions can collect in the risk assessment is very limited. Therefore, investing institutions usually determine the most cost-effective way after balancing their costs and benefits, i.e., long-term cooperation with the owner of the information. This has caused the monopoly phenomenon of information to become increasingly serious and also led to an increase in information asymmetry (Wu et al., 2018; Li et al., 2019).

### Solutions for Information Asymmetry

Although information asymmetry exists objectively and it is very difficult to avoid, the information asymmetry problem will lead to the problem of adverse selection and moral problems. Accordingly, it is urgent for all parties to weaken the information asymmetry. Adverse selection in two directions proposes ways to weaken the information asymmetry problem; however, it is worth mentioning that the information asymmetry problem can only be weakened to a certain extent and cannot be completely eliminated.

(1) Adverse selection: It refers to a situation in which the party with less information is confused or even deceived by the party with more information to gain more benefits. Therefore, the root cause of the adverse selection problem is the amount of information obtained. Therefore, reducing the cost of obtaining information and expanding the channels of obtaining information can solve the negative impact of adverse selection to a certain extent.

Because each venture capital institution has its private information, if the information of venture capital institutions can be integrated, it will greatly reduce the cost of collecting information and improve the efficiency of each investing institution. In the meantime, if an intermediary conducts information verification or adjustment, it will greatly increase the efficiency of information circulation. However, China’s current risk investing institutions are characterized by low service capacity, low service quality, and poor professionalism. As a result, it is also crucial for the selection of intermediary personnel (Zheng and Liu, 2020).

The method for investors to screen information will also have a greater impact on the flow of information. At present, the method for screening information by investing institutions is only internal screening. If the information screening method of investing institutions can be changed to telephone consultation, face-to-face consultation, and obtaining evidence from other practitioners, it will greatly improve the efficiency of information screening, thereby accelerating the flow of information and expanding the information transmission path (Chen, 2019).

(2) Moral hazard: To gain more benefits, the agent of the information superior party usually makes a profit by hiding part of the information, which will cause great distress to the information inferior party principal and lose the interests of the principal.

As a result, the restraint measures for both the agent and the client will play an important role, and binding the signing of the contract can protect the interests of the client to the greatest extent.

For investing institutions and investors, they may choose to renew the contract to restrict investing institutions. If the investing institution has hidden information or deceptive behaviors during this period, the contract will be terminated and the investing institution may be subject to legal sanctions. Investing institutions and investors can be required to invest together so that investment agencies will be more responsible for investment projects. The investor can conduct regular or irregular evaluations of the investing institutions. In case of any behavior endangering the interests of the investor, the investor has the right to immediately terminate the contract and apply for compensation. After the investment is over, both parties should carry out thorough interests and division of power to ensure the interests of both parties. Also, the two parties can sign some substantial reward regulations, such as the investor’s income directly determines the investing institution’s income, and the investor introduces the corresponding client to the investing institution (Rogoza et al., 2018; Zhu et al., 2019).

For investing institutions and entrepreneurs, they may choose to invest gradually to protect the interests of investors. Entrepreneurs can be asked to list the flow of funds, and investing institutions can be asked to list what can and cannot be done during the contract period, providing a guarantee for both parties. A thorough investigation of the invested enterprise is conducted, especially its capital structure, and clarifying the capital structure is conducive to the investing institution’s designation of investment plans or whether to invest.

Liability risk is also one of the factors that must be considered in moral hazard. Therefore, when concluding the corresponding delegation or investment contract, the person responsible should be defined. Also, investing institutions may reduce the risks by joining the board of directors, participating in financial activities, and handling crisis events to lower the investment risks.

### Research Samples and Data

In this study, the enterprises listed on the Shanghai and Shenzhen stock exchanges between 2014 and 2018 were taken as the research objects, and the financial data of these enterprises were sorted out for research and analysis. The data were mainly sourced from the CSMAR database. Data with poor comparability, cross-listing data, missing data, and corporate data from the financial industry were eliminated. The STATA was used to conduct a retrospective analysis of the data selected (Shen and Ho, 2020). In addition, a total of 133 enterprises were included, all of which were small and medium-sized enterprises.

### Research Model Design

Based on the previous explorations, the residual model was used to study the information asymmetry and investment efficiency of enterprises in this research. The model design is as follows:

(1) $$\begin{array}{ll} Inv_{it} & ={\text{β}}_{0}+{\text{β}}_{1}Growth_{it-1}+{\text{β}}_{\text{2}}Lev_{it-1}+{\text{β}}_{\text{3}}Cash_{it-1}\\ & +{\text{β}}_{4}Age_{it-1}+{\text{β}}_{5}Size_{it-1}+{\text{β}}_{6}Ret_{it-1}+{\text{β}}_{\text{7}}Inv_{it-1}\\ & +\sum Yearindicator+e_{it} \end{array}$$

where *Inv*_*it*_ refers to the total investment obtained by an enterprise in *t* years. To ensure the accuracy of the data, the newly added total investment is expressed as the difference between “cash paid for the purchase and construction of fixed assets, intangible assets, and other assets” and “cash received from the disposal of fixed assets, intangible assets, and other long-term assets.” The corresponding standardized processing is performed in the model.

*Growth*_*it–1*_refers to all the growth opportunities that the enterprise has in the previous year. The growth opportunities of the enterprise are directly proportional to the amount of investment the enterprise receives and will affect the amount of new investment to a certain extent. Therefore, this study correlates the expected investment volume with the growth opportunities of the enterprise in the previous year. *Lev*_*it–1*_refers to the asset-liability ratio of an enterprise in the previous year. The increase in the asset-liability ratio represents the increase in the negative impact of the enterprise on the borrower; therefore, the cost of obtaining investment for an enterprise will also increase, and the asset-liability ratio will have a negative correlation with the amount of new investment. *Cash*_*it–1*_represents the cash holdings of an enterprise in the previous year, which is directly proportional to the new investment. *Age*_*it–1*_represents the listing period of an enterprise as of the previous year, which is negatively related to the new investment. *Size*_*it–1*_represents the scale of an enterprise in the previous year, which is in a positive correlation with new investment volume. *Ret*_*it–1*_represents the stock return of an enterprise, which is positively correlated with the new investment volume. *Inv*_*it–1*_represents the new investment volume of the previous year. ε_*it*_is used to measure the investment situation of the enterprise.

The model for measuring the impact of information asymmetry on enterprise investment efficiency is as follows:

(2) $$\begin{array}{ll} Inef_{it} & ={\text{β}}_{0}+{\text{β}}_{1}IA_{it}+{\text{β}}_{\text{2}}Size_{it-1}+{\text{β}}_{\text{3}}Fcf_{it-1}+{\text{β}}_{\text{4}}Q_{it-1}\\ & +{\text{β}}_{5}Qx_{it-1}+{\text{β}}_{6}DS_{it-1}+{\text{β}}_{7}JS_{it-1}\\ & +{\text{β}}_{8}DL_{it-1}+\sum Year \end{array}$$

where *Inef* represents the investment efficiency of the enterprise; *IA* represents the information asymmetry; Size represents the size of the enterprise; *F*_*cf*_ represents the internal cash flow; *Q* represents the growth opportunity of the enterprise; *QX* represents the nature of the enterprise; *DS* represents the size of the board; *JS* represents the size of the supervisory board; *DL* represents the proportion of independent directors; and Year represents the annual control variable.

## Results

### Analysis Results of Investment Efficiency Model

**FIGURE 1:**
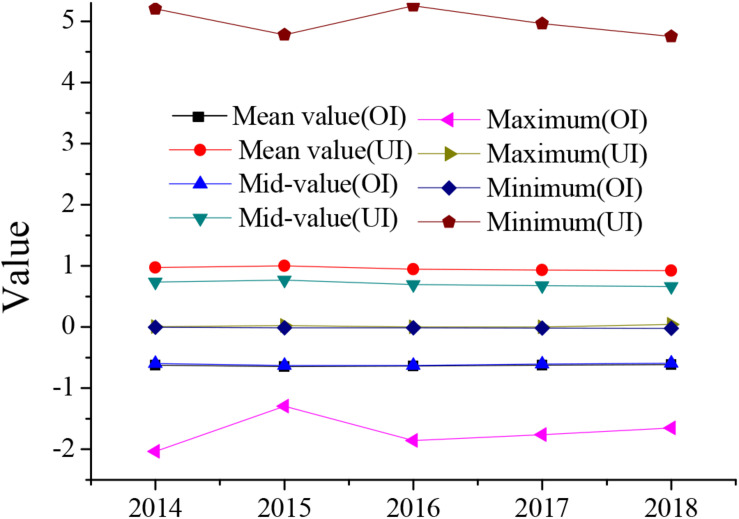
Investment efficiency statistics.

Descriptive statistics of the investment efficiency of the enterprises studied in this study are shown in Figure 1.

In Figure 1, OI indicates over investment and UI indicates under-investment. The situation in which Chinese enterprises have obtained investment has shifted from under-investment to over investment. Listed enterprises have over-funded but are cautious in investing, causing huge economic losses.

After analysis of the proposed investment efficiency model, the parameters of the selected enterprise are shown in Table 1.

**TABLE 1:** Description of investment efficiency.

**Variable name**	**Minimum value**	**Maximum value**	**Median**	**Standard deviation**	**Mean value**
*Inv*_*it–1*_	−3.5421	9.7340	0.0475	0.2217	0.0941
*Growth*_*it–1*_	−1.0026	81.9011	0.1393	2.2337	0.2973
*Lev*_*it–1*_	0.0078	8.2613	0.5103	0.4421	0.5016
*Cash*_*it–1*_	−0.03511	31.6032	0.1371	1.1725	0.3035
*Ret*_*it–1*_	−1.0042	1.7054	0.0201	0.4032	0.0402
*Size*_*it–1*_	16.7235	28.3973	22.022	1.4403	22.0046
*Inv*_*it*_	−0.7149	9.7133	0.057	0.3044	0.0862

As shown in Table 1, the investment level of the enterprises listed on the Shanghai and Shenzhen stock exchanges selected in the study from 2014 to 2018 is below 7.69%. The investment level is low, and the difference between the minimum and maximum of the new investment volume is 10.4282, which is very huge, indicating that the investment level between enterprises is huge. In addition, for the growth opportunities of enterprises, the difference between the minimum value −1.0026 and the maximum value 81.9011 is too large. It shows that there is a serious difference in growth opportunities between enterprises. The difference between cash holdings, stock returns, and company size is also large. This shows that the companies are at different levels and the amount of investment they receive will also be at different levels.

### Results of Regression Analysis on the Impact of Information Asymmetry on Investment Efficiency

The above analysis shows that the investment efficiency of China is at a relatively low level. STATA software was used to test and analyze information asymmetry and enterprise investment efficiency. The analysis results are shown in Table 2.

**TABLE 2:** Regression analysis of the impact of information asymmetry on investment efficiency.

**Variable name**	**Coefficient**	**Standard deviation**	***T*-value**	***p*-value**
Constant	0.9413***	0.2031	4.31	0.000
*IA*_*it*_	0.0119**	0.0029	2.35	0.019
*Size*_*it–1*_	−0.0596***	0.0070	−8.03	0.000
*Fcf*_*it–1*_	0.0018	0.0018	0.99	0.320
*Q*_*it–1*_	−0.0058	0.0091	−0.71	0.497
*QX*_*it–1*_	0.0117	0.0261	0.45	0.661
*DS*_*it–1*_	0.0229***	0.0068	4.01	0.000
*JS*_*it–1*_	0.0053	0.0139	0.53	0.601
*DL*_*it–1*_	0.7694***	0.2011	3.81	0.000
*Year*	Control	Control	Control	Control

*** and ** represented significance at the 1% and 5% levels.

As shown in Table 2, there is a significant correlation between information asymmetry and investment efficiency, and the regression coefficient is 0.0119. This shows that the investment efficiency of an enterprise is correlated with the degree of information asymmetry. The more serious the information asymmetry is, the lower the investment efficiency of the enterprise is. Table 2 also shows that the larger the scale of the enterprise is, the higher the investment efficiency of the enterprise is. The possible reason is that larger enterprises pay more attention to information asymmetry, and the quality of their information is higher; thus, investment institutions can obtain effective information, thereby increasing the competitiveness of enterprises. Also, large-scale enterprises will pay more attention to their images, which will reduce information confusion or concealment. Besides, as shown in Table 2, the higher the proportion of independent directors is, the worse the investment effect of the enterprises is.

### Case Study 1

**FIGURE 2:**
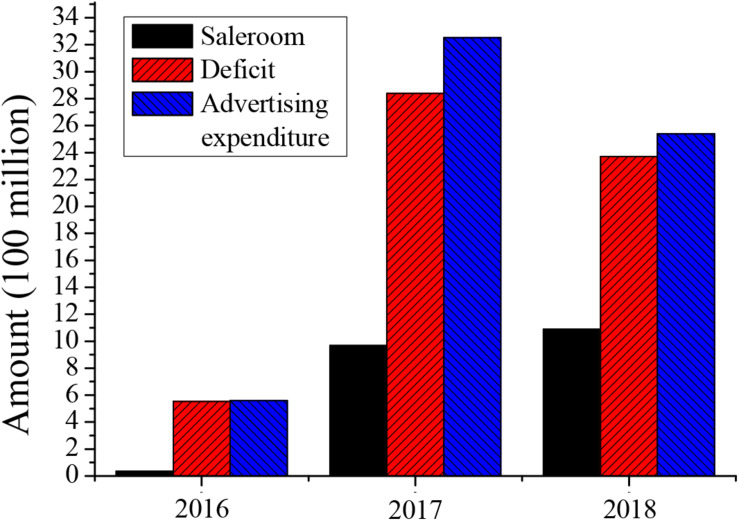
Comparison of sales and losses and advertising investment of an enterprise.

Taking an enterprise as an example, this study conducted a comparison of sales and losses and advertising investment, as shown in Figure 2.

According to Figure 2, the operating income of this enterprise is not optimistic, and excessive advertising investment has caused it to suffer serious losses. Therefore, this shows that there is a certain problem in the information collected before decision-making. The adverse selection has already occurred before this enterprise made investment decisions. The major reason for its mistakes is that the market investigation is not careful, and its incomplete understanding of the market caused serious losses.

For the problem of adverse selection, this enterprise should immediately begin to comprehensively collect information, improve the part of the market research that has been not carefully done before, and reform the brand to a certain extent, thereby finding the brand positioning rather than whimsical. Second, the reputation of the brand is also an important resource. Thus, the brand should strengthen brand management and carry out brand promotion according to the actual needs of consumers.

For the problem of moral hazard, the behavior of concealing information has caused the credibility of the brand to plummet. In the early stages of the brand’s existence, the price of the brand has varied, and the online price has been lower than the offline price. Therefore, the reputation of the brand is reduced. The brand should first price uniformly, and the brand information should also be announced and disclosed in time to restore the brand image.

### Case Study 2

Due to the rapid progress of the coastal economic belt, an old industrial base has gradually declined. Most of the existing industries are intensive industries, and there is an urgent need to upgrade the industrial structure. However, there is not enough driving force for the development of high-tech industries and the tertiary industry. Although the investment environment has been greatly improved with the efforts of this industrial base, for investment enterprises, there is a relatively serious information asymmetry between the actual environment and the investment environment. Therefore, there is no large-scale capital entry.

Under such circumstances, the industrial base selected two cities for the pilot operation to develop enterprise venture capital. Despite the relatively significant results, compared with other provinces and cities across the country, the investment situation is still lagging.

Because of the remote geographical environment of the base and the short development time of venture capital, the base has not reached an effective level in handling and managing information asymmetry. In addition, the economy of this base is relatively backward; thus, entrepreneurs will make mistakes such as exaggerating the enterprise level or concealing the actual situation of the enterprise if they want to get investment. However, the investing institutions in this area are not capable of screening information and the information transmission is not smooth, which makes it more difficult for investing institutions to choose enterprises. Therefore, the situation of adverse selection occurs, and it is difficult to solve the moral hazard.

To solve the above-mentioned practical problems of morality at this base, the government has carried out the following substantive measures: (1) Material incentives: The interests of entrepreneurs, funders, investing institutions, technical talents, and other related personnel are linked, and all parties are inspired to make efforts that are conducive to the development of the enterprise. (2) Reputation incentives: Investing institutions draw investment for enterprises based on their reputation, and the investment they receive will be directly related to their reputation. (3) Encouragement of control rights: Entrepreneurs are granted with specific control rights to meet the individual rights of entrepreneurs. The self-worth of entrepreneurs is realized, and the size of control rights is linked to the state of business operations, thereby encouraging entrepreneurs to work hard. (4) Stock option incentives: The stock options of enterprises are connected with managers, entrepreneurs, corporate employees, and investors to encourage all parties to do their jobs.

## Discussion

The ability to invest institutions, funders, and entrepreneurs has certain differences in their ability to obtain information. Also, they have different directions and major concerns about information collection. Therefore, the information asymmetry truly exists, which will become a factor that will bother the three parties. The weakening of information asymmetry and even its solution is the focus of attention of all sectors of society. Based on the regression summary of research results worldwide, a theoretical analysis is performed, the hypotheses are proposed, and empirical researches are conducted. The information asymmetry mainly refers to the information asymmetry existing between the investor and the financier (Samet and Jarboui, 2017). Specifically, investors are often in a disadvantaged information position that does not have enough information about investment projects. At this time, an average evaluation method will be adopted. However, such an average evaluation method often leads to the decline of the enterprise investment efficiency (Wu et al., 2020).

Through research, this study finds a significant correlation between information asymmetry and investment efficiency, with a regression coefficient of 0.0119. It indicates that there is a negative correlation between the investment efficiency of enterprises and the degree of information asymmetry. The more serious the information asymmetry problem is, the lower the investment efficiency of the enterprise is; the larger the scale of the enterprise is, the higher the investment efficiency of the enterprise is. These results are consistent with the research results of Chen et al. (2018).

The information asymmetry exists objectively. In order to reduce the impact of the information asymmetry problem, it is necessary to improve the investment efficiency of the enterprises. For investment institutions, they should increase their sense of responsibility in publishing real information, and corresponding reward and punishment measures may be formulated. The higher the quality of the information the institution publishes, the more generous the rewards it receives. Otherwise, the institution should bear the corresponding punishment. For the enterprises, if the manager has entrepreneurial psychology, carefully manages the enterprise, and uses a positive method to make the enterprise develop rapidly, the entrepreneur will be given a corresponding reputation incentive. For investors, they should have their investment vision and avoid following others blindly.

## Conclusion

This study found that when both investing institutions and investors have entrepreneurial psychology, they will consider the problem according to the entrepreneur’s way of thinking. Thus, they will have more considerations on adverse selection and human factors. When they have a consistent opinion, the occurrence of information asymmetry may be avoided, and the investment risks may be reduced.

Although some achievements have been made in this study, there are still the following limitations:

(1) The sample size in this study can be expanded further. The data were selected from listed enterprises from 2014 to 2018 and it did not cover other time frames and the situation of other enterprises, leading to certain limitations and insufficient sample interval.
(2) The residual model used in this study was researched and designed by non-Chinese scholars, which may not fully comply with China’s national conditions.

Although Chinese academics believe that the model may well simulate China’s investment market, it still has certain one-sidedness. Therefore, long-term large-scale information asymmetry needs to be further studied to accommodate different time frames and different development stages of enterprises.

## Data Availability Statement

The raw data supporting the conclusions of this article will be made available by the authors, without undue reservation.

## Ethics Statement

The studies involving human participants were reviewed and approved by the Shaanxi University of Science and Technology. The patients/participants provided their written informed consent to participate in this study.

## Author Contributions

PD wrote the manuscript. HS and ZX made the review. All authors contributed to the article and approved the submitted version.

## Conflict of Interest

ZX was employed by Maolong International Financial Leasing Co., Ltd. The remaining authors declare that the research was conducted in the absence of any commercial or financial relationships that could be construed as a potential conflict of interest.
